# Highly dangerous road hazards are not immune from the low prevalence effect

**DOI:** 10.1186/s41235-024-00531-3

**Published:** 2024-02-02

**Authors:** Jiali Song, Benjamin Wolfe

**Affiliations:** Department of Psychology, University of Toronto Mississauga, 3359 Mississauga Rd, Mississauga, ON L5L 1C6 Canada

## Abstract

The low prevalence effect (LPE) is a cognitive limitation commonly found in visual search tasks, in which observers miss rare targets. Drivers looking for road hazards are also subject to the LPE. However, not all road hazards are equal; a paper bag floating down the road is much less dangerous than a rampaging moose. Here, we asked whether perceived hazardousness modulated the LPE. To examine this, we took a dataset in which 48 raters assessed the perceived dangerousness of hazards in recorded road videos (Song et al. in Behav Res Methods, 2023. 10.3758/s13428-023-02299-8) and correlated the ratings with data from a hazard detection task using the same stimuli with varying hazard prevalence rates (Kosovicheva et al. in Psychon Bull Rev 30(1):212–223, 2023. 10.3758/s13423-022-02159-0). We found that while hazard detectability increased monotonically with hazardousness ratings, the LPE was comparable across perceived hazardousness levels. Our findings are consistent with the decision criterion account of the LPE, in which target rarity induces a conservative shift in criterion. Importantly, feedback was necessary for a large and consistent LPE; when participants were not given feedback about their accuracy, the most dangerous hazards showed a non-significant LPE. However, eliminating feedback was not enough to induce the opposite of the LPE—prevalence induced concept change (Levari et al. in Science 360(6396):1465–1467, 2018. 10.1126/science.aap8731), in which participants adopt a more liberal criterion when instances of a category become rare. Our results suggest that the road hazard LPE may be somewhat affected by the inherent variability of driving situations, but is still observed for highly dangerous hazards.

**Supplementary Information:**

The online version contains supplementary material available at 10.1186/s41235-024-00531-3.

## Significance statement

When driving, the most dangerous situations that we must avoid (collisions and near collisions) are very rare. The low prevalence effect (LPE) is a cognitive limitation commonly found in search tasks, in which observers miss infrequent targets. Previously, we have shown that the LPE also applies to road hazards: when viewing videos of road situations, drivers miss rare hazards (Kosovicheva et al., 2023). However, some road hazards are perceived as more dangerous than others. To understand this variation in road hazards, we asked a separate sample of 48 drivers to assess the hazardousness of each road video on a continuous scale (Song et al., 2023). These ratings indicate that perceived hazardousness is more granular than a simple present-absent judgement. This study combines these two datasets to examine whether perceived hazardousness modulates the LPE. One may think, because some road hazards are extremely dangerous, that they would capture attention and therefore be immune to the LPE. Our data suggests that this is not the case, and that even extremely dangerous hazards are subject to the LPE. Observers are more likely to miss rare hazards regardless of how dangerous they are. These results suggests that, as road safety intervention programs, such as Vision Zero, make hazards rarer, the LPE may exacerbate the effects of the remaining hazards. Special attention should be paid to rare hazards that are particularly dangerous in efforts to reduce road hazard incidence and impact.

## Introduction

A key cognitive limitation that impairs the detection of rare targets is the low prevalence effect (LPE), a well-known effect in the visual search literature (Wolfe et al., 2005), in which observers are more likely to miss targets when they are rare, compared to when they appear frequently. This effect has been observed in various high stakes contexts, such as medical image assessment and airport baggage search, as well as in the laboratory (Buser et al., 2020; Evans et al., 2013; Horowitz, 2017; Wolfe et al., 2005, 2007).

Much like rare search targets (e.g., weapons in luggage), road hazards occur relatively seldom in real driving situations. However, driving differs from a typical visual search task in several ways. Road situations are dynamic, and hazards can unfold over a split second, resulting in extremely high time pressure for responding safely (Green, 2000; Wolfe et al., 2019, 2020) and the consequences of these actions are immediate. In contrast, in other contexts such as looking for a tumor in a mammogram, search is often untimed, and the consequences of a decision may only be known years later. As a result, findings from static visual search tasks may not apply to dynamic road situations.

Nevertheless, previous studies investigating low prevalence effects in driving contexts have found a higher probability of missing rare road hazards compared to common ones (Beanland et al., 2014; Kosovicheva et al., 2023). In a simulated driving study, vehicle types that appeared relatively rarely were missed more often compared to vehicles that appeared more frequently (Beanland et al., 2014). The LPE also occurs when varying the overall prevalence of hazards in naturalistic hazard videos, as observers missed road hazards twice as often when they were rare compared to when they appeared more frequently (Kosovicheva et al., 2023). However, perceiving hazards on the road is not a simple binary hazard present/absent judgment. A paper bag on the road is not dangerous, whereas a rampaging moose is very dangerous. Does this variation in hazardousness modulate the LPE?

There are several reasons to expect hazardousness to modulate the LPE. One potential source of misses in the LPE is the failure to attend to the target even when it was visible. However, highly dangerous hazards may be easier to attend to than less dangerous hazards because attention can be biased towards threatening stimuli (Fabio & Caprì, 2019; Mulckhuyse & Dalmaijer, 2016; Schmidt et al., 2015). One possibility is that hazards believed to be dangerous might capture attention and be less subject, or even immune, to the LPE.

A second source of error that may contribute here is that prevalence changes what observers are willing to categorize as a target. Signal detection analyses show a more conservative criterion when prevalence is low than when prevalence is high (Horowitz, 2017; Kosovicheva et al., 2023; Wolfe et al., 2007). In a driving context, if participants adopt a more conservative criterion when hazards are rare, then a hazard would need to be more dangerous before participants are willing to categorize it as such.

In other circumstances, observers may show the opposite of the LPE: prevalence induced concept change (PICC; Levari, 2022; Levari et al., 2018; Lyu et al., 2021). PICC occurs when participants are asked to categorize individual stimuli drawn from a continuous space. As instances of the target category become rare, the criterion becomes more liberal. However, this effect depends on the presence of feedback on response accuracy. PICC occurs when feedback is absent, whereas LPE occurs when feedback is present (Lyu et al., 2021). When perceiving road hazards, we may expect a more liberal shift in criterion when feedback is absent, in line with PICC. Although classic signal detection analyses found a reduced LPE but not PICC in previous work (Kosovicheva et al., 2023), it is possible that PICC may emerge when perceived hazardousness is accounted for.

The stimuli used in Kosovicheva et al., 2023 came from the Road Hazard Stimulus set (Wolfe et al., 2020), which has since been expanded to include ratings of hazardousness for each video clip from 48 independent raters (Song et al., 2023). Previously, we showed that drivers can accurately judge the relative hazardousness of these situations on a continuous scale with brief, 333 ms, glimpses of the road (Song et al., 2023). Moreover, consistent with the idea that hazardousness assessments are more granular than a simple present/absent binary judgment, participants’ ratings were distributed along the entire scale.

Since the stimuli are identical across these two studies (Kosovicheva et al., 2023; Song et al., 2023), we can analyze the hazard detection data from Kosovicheva et al. (2023) together with the rating data from Song et al. (2023) to determine whether the LPE for road videos varies with their perceived hazardousness. Specifically, the current study investigated three questions: (1) Are hazard detection rates related to the perceived dangerousness of hazards? (2) Are extremely dangerous hazards immune to the LPE?, and (3) Does trial-wise feedback modulate the minimum dangerousness participants need to categorize a video as a hazard?

## Methods

### Hazard detection data (Kosovicheva et al., 2023)

**Fig. 1:**
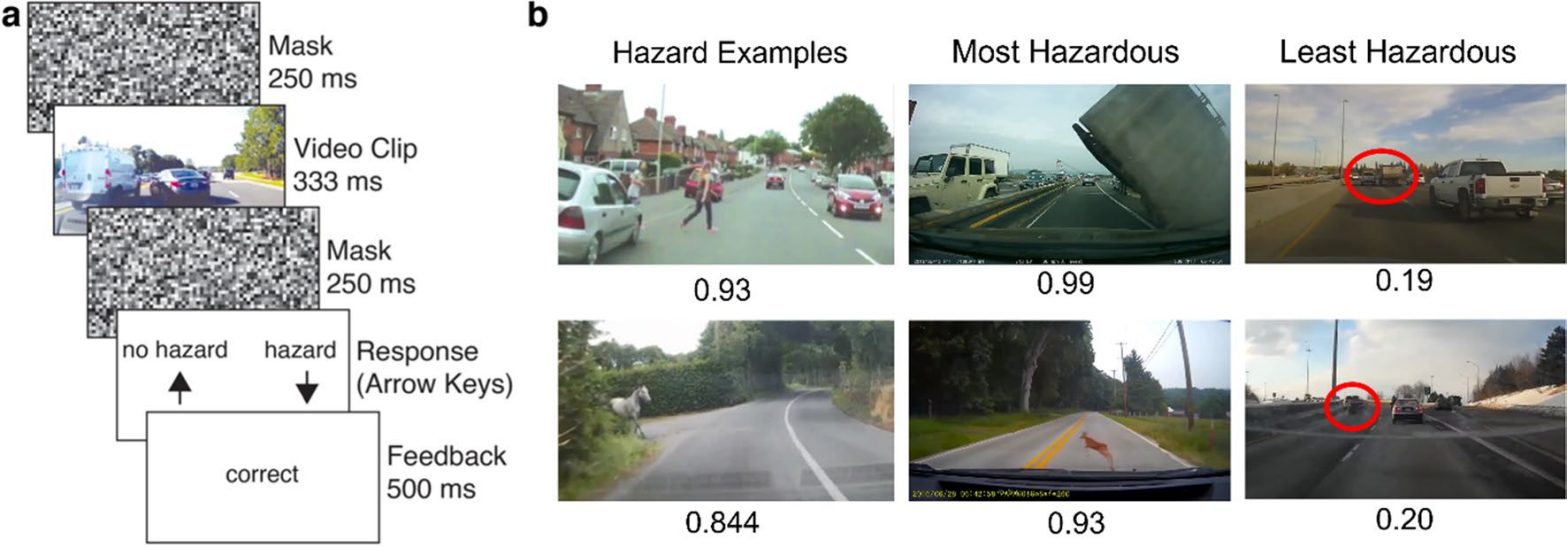
A schematic of the paradigm from Kosovicheva et al., 2023. First published in *Psychonomic Bulletin & Review,* **30**, 212–223, 2023 by Springer Nature. **a** On each trial, participants watched a segment of video footage of a real road scene taken from a front-facing dashboard camera. The video was preceded and followed by a randomly generated noise mask. Following the second mask, participants indicated whether there was a hazard that required an immediate evasive response. After the response, feedback was provided based on response accuracy. **b** Examples of hazards in the stimulus set shown to participants. Hazards included (but were not limited to) vehicles, pedestrians, and animals. The median hazardousness rating is shown below each image. For least hazardous videos, the hazards are indicated by red circles for illustration. Red circles were not shown in the experiment

The hazard detection data came from a previously published study that measured the low prevalence effect (LPE) across five online experiments. Participants (n = 16 per experiment) watched brief road videos (333 ms) from the Road Hazard Stimulus set (Wolfe et al., 2020), a collection of videos sourced from online social media (see Fig. 1 for examples). Participants indicated whether each video contained a hazardous situation requiring an immediate response to avoid a collision (‘hazard’ vs. ‘no hazard’). In each experiment, hazard prevalence was manipulated within-groups in two separate sessions, with a high (50%) and a low (4%) prevalence session (except in experiment 5, which had rates of 10% and 1%). Participants completed 440 trials in the high prevalence condition and 500 trials in the low prevalence condition (480 and 580 trials, respectively, for experiment 5). Trial numbers differed due to the limited number of available videos. Session order was counterbalanced across participants. Hazard-present and hazard-absent videos were randomly drawn without replacement from 432 videos annotated as hazard-present and 924 videos annotated as hazard absent (1376 videos in total).

In experiment 1 (full feedback), feedback regarding response accuracy (correct vs. incorrect) was provided at the end of each trial. In experiment 2 (no feedback), feedback was not given. In experiment 3 (partial feedback), participants only received feedback if they missed a hazard. In experiment 4 (response correction), participants were allowed to correct their response to account for possible motor errors before receiving feedback. In experiment 5 (lower prevalence), to better match the range of hazard prevalence rates in real-world driving, hazard prevalence in high and low conditions were lowered to 10% and 1%, respectively. Experiment 5 also included an instruction manipulation where half the participants were briefed about the LPE before the experiment and the other half was not (n = 16 per group, n = 32 total). Since the briefing did not affect the LPE, these groups have been combined here.

All participants were, by self-report, aged 20 and 35 years old, in possession of a valid driving license, had normal or corrected to normal visual acuity, and were residents in Canada, the United States of America, or the United Kingdom. Across all five experiments, participants had a mean age of 27 (*SD* = 4.4) years.

### Hazard rating data (Song et al., 2023)

The hazard ratings data were collected previously as a part of the Road Hazard Stimuli dataset (Song et al., 2023). To obtain assessments of perceived dangerousness for each video clip, 48 licensed drivers (aged 20–35, mean = 29, *SD* = 4.35) who had not previously seen the stimulus videos were recruited through Prolific. The inclusion criteria were identical as that of Kosovicheva et al., 2023. Each participant rated the hazardousness of the full set of 1376 road videos (from the dataset described above) on a continuous scale between 0 (very safe) and 1 (very hazardous). After viewing each video clip, participants recorded their untimed response by adjusting the position of a slider. Videos were rated in two sessions lasting approximately 45 min each. Each participant rated all videos from the dataset in a random order. Participants were not informed about the hazard-absent vs. hazard-present classification of each video.

### Data analysis

We extracted the proportion of “hazard present” responses for each movie from each experiment in the dataset, and the median hazardousness of each movie clip from the hazard rating data. To examine the relationship between the probability of “hazard present” response and hazardousness ratings, we used a generalized linear mixed effects regression model to fit a binomial distribution to the data across all participants (see Additional file 1: Table S1 for details). There were insufficient trials to fit a model at the individual participant level, particularly for the low-prevalence conditions. The dependent variable was the proportion of yes responses for all movies, and the predictors were median ratings for each movie, prevalence (high or low), experiment, and all interactions involving median ratings. Participant was included as a random variable.

To determine the effect of prevalence on participants’ hazard detection responses, we extracted the 50% threshold (the hazardousness level at which participants were equally likely to indicate hazard present and hazard absent) from the fitted psychometric functions. We calculated the difference between thresholds in the high and low prevalence conditions for each experiment to examine whether the results were consistent with the LPE (higher thresholds under low prevalence) or PICC (lower thresholds under low prevalence). To quantitatively determine whether this difference was significantly different from zero, we conducted permutation tests by shuffling the prevalence condition labels within participants and calculating the difference in thresholds while preserving within-subjects effects over 1000 iterations. This procedure generated separate null distributions against which we compared each observed threshold to calculate the resulting *p*-values.

Next, to determine whether the LPE varied with perceived hazardousness, we restricted the analyses to only hazard-present videos (based on the hazard present vs. absent classification in Song et al., 2023 and Wolfe et al., 2020), divided into quartiles based on median hazardousness. We calculated the magnitude of the LPE by calculating the difference in miss rate between the low and high prevalence conditions, such that more positive values indicate that participants were more likely to miss hazards under low prevalence. We used permutation tests to determine whether the LPE differs from zero in each quartile by shuffling the prevalence condition labels within each participant and quartile over 1000 iterations. This procedure was done separately for each experiment.

We also used permutation tests to compare whether the LPE in the top quartile differed from other quartiles separately for each experiment. To do so, we calculated the LPE using only videos from the top quartile for each participant. Then, we generated a null distribution by calculating the LPE after shuffling the quartile labels within each participant for 1000 iterations, which provided *p*-values for the observed LPE of each quartile while maintaining subject-level effects.

## Results

**Fig. 2:**
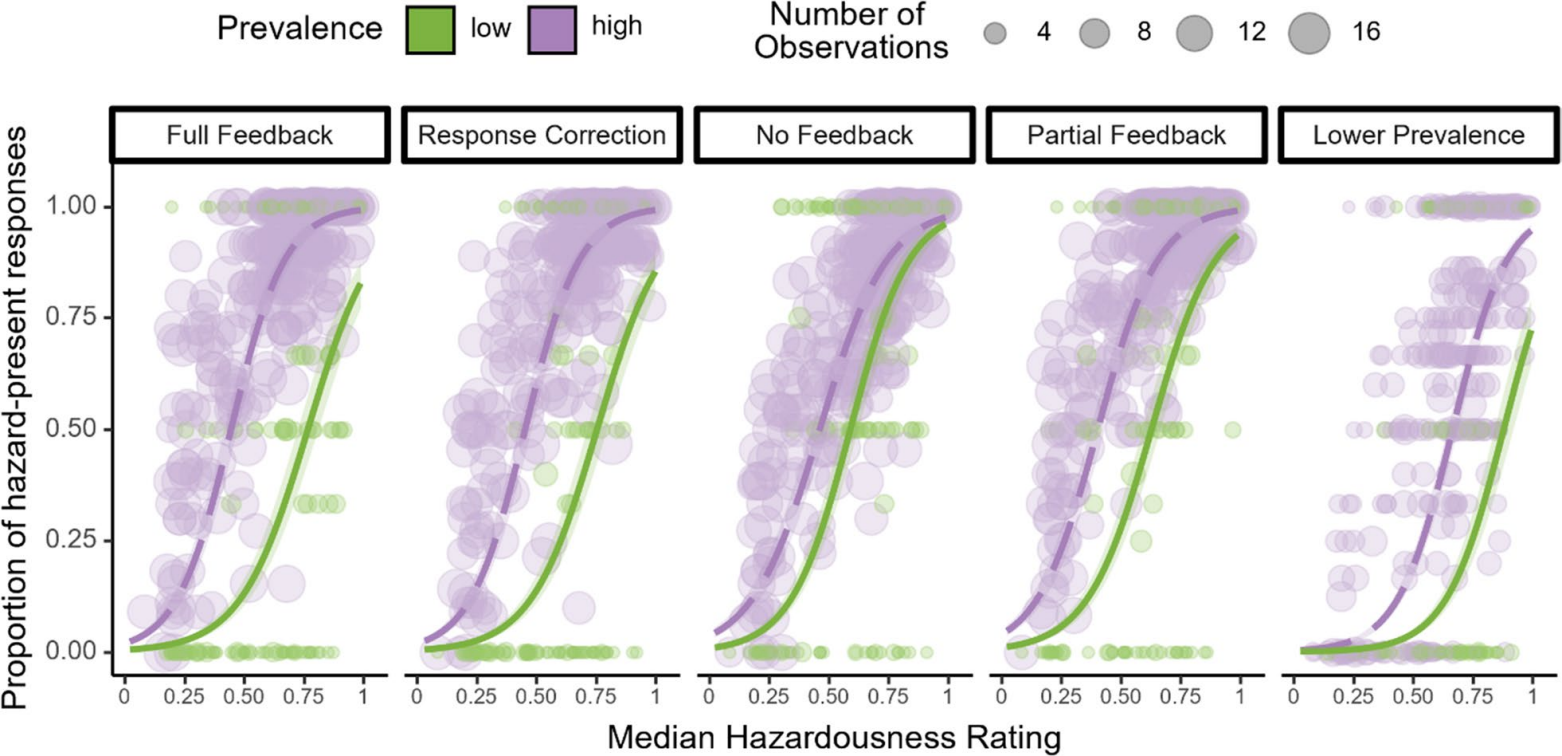
Proportion of “hazard-present” responses as a function of median hazardousness ratings for each movie (represented by each circle). Across all experiments, the probability of a “hazard-present” response increased as videos became more dangerous. Consistent with the LPE, there were fewer “hazard-present” responses under low hazard prevalence (green) compared to high hazard prevalence (purple) across all hazardousness levels. Interestingly, this pattern was observed even in the no feedback experiment, although the magnitude of the LPE is much smaller than when feedback was present. These results are consistent with previous work that found that low hazard prevalence induces a more conservative criterion, such that participants needed the video to be more hazardous before they are willing to respond “hazard-present” (e.g. Wolfe et al., 2007)

The results of the logistic regressions investigating the effect of hazardousness ratings on hazard detection (Fig. 2) showed that across experiments, the probability of a “hazard present” response increased with median hazardousness rating, suggesting that participants are sensitive to variations in dangerousness of hazards. We found that an increase of 0.1 in median hazard rating increased the probability of a “hazard-present” response by 0.87 logit units (Standard Error = 0.16032, *z* = 54.524, *p* < 0.001). Furthermore, Type III Wald *χ*^2^ tests found a significant three-way hazardousness rating, prevalence, and experiment interaction (*χ*^2^ (4) = 222.846, *p* < 0.001), indicating that the LPE was affected by both experiment and hazardousness rating.

Given the significant interaction with Experiment, we examined the LPE separately for each experiment for a more detailed analysis. Interestingly, statistically significant LPEs were found for all 5 experiments (*χ*^2^ (1) ≥ 32.23, *p* ≤ 0.009; see Table 1). Even when there was no feedback, there was a smaller, but significant LPE (*χ*^2^(1) = 37.49, *p* < 0.001).

**Table 1:** Results of permutation tests for each individual experiment and quartile

Experiment	LPE (Threshold)	*p*	LPE Q1	*p*	LPE Q2	*p*	LPE Q3	*p*	LPE Q4	*p*
Full Feedback	0.30	< 0.001	0.32	< 0.001*	0.2	< 0.001*	0.21	< 0.001*	0.12	< 0.001*
Response Correction	0.28	< 0.001	0.37	< 0.001*	0.26	< 0.001*	0.18	< 0.001*	0.06	0.02
No Feedback	0.13	< 0.001	0.09	0.13	< 0.001	0.99	0.04	0.25	0.06	0.02
Partial Feedback	0.23	< 0.001	0.25	< 0.001*	0.09	0.05	0.16	< 0.001*	0.04	0.05
Lower Prevalence	0.32	< 0.001	0.20	0.011	0.16	0.05	0.36	< 0.001*	0.12	0.09

All LPE measures were calculated such that the more positive the value, the higher the miss rate was under low prevalence compared to high prevalence. The leftmost column shows the overall LPE calculated as the shift in threshold, and the remaining columns show the difference in miss rates between low and high prevalence conditions separately for each hazard quartile (Q1: least hazardous quartile; Q4: most hazardous quartile)

*Significant based on Bonferroni-corrected critical alpha was 0.0025

To further investigate the LPE in each experiment, we also examined the shift in the threshold of the psychometric function in each experiment separately. Figure 2 shows a rightward shift in the estimated thresholds under low prevalence compared to high prevalence. The results of permutation tests conducted on the fitted parameters were consistent with the observation that participants were less likely to indicate “hazard present” under low prevalence (see Table 1).

**Fig. 3:**
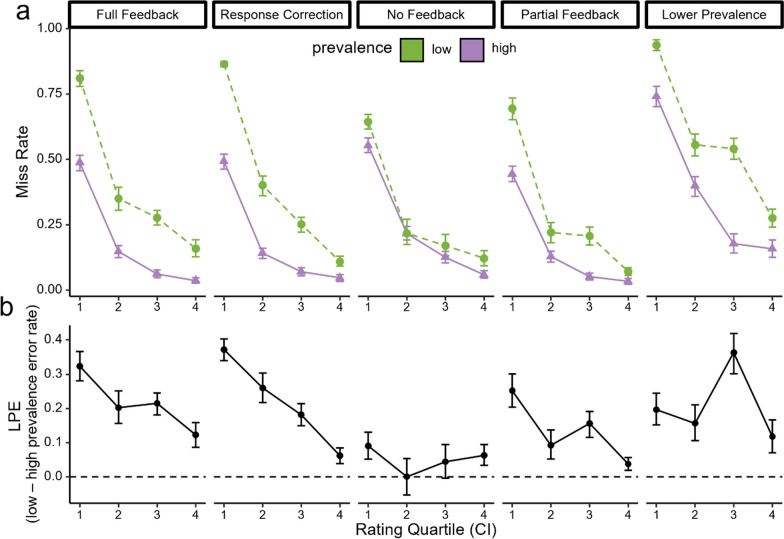
**a** Illustrates miss rate as a function of rating quantile and prevalence, with green and purple representing the low and high prevalence conditions, respectively. **b** illustrates the LPE as a function of quartile, calculated by subtracting miss rates in the high prevalence condition from the low prevalence condition. The dashed horizontal line represents no LPE. In both figures, each panel represents an experiment, and error bars represent the bootstrapped 95% confidence intervals. Similar to results of the logistic regression, miss rates decreased as hazardousness ratings increased. Although the magnitude of the LPE for the most hazardous quartile (Q4) is smaller than the least hazardous quartile (Q1), the miss rates under low prevalence remain approximately twice as high as miss rates under high prevalence condition (see Additional file 1: Fig. S1). Moreover, removing feedback decreased the LPE such that the LPE was not significantly different from zero at any quartile, and there was no evidence of PICC. However, there was a significant LPE overall, but it may be smaller than what can be detected with the decreased power in each quartile

Based on visual examination of Fig. 3a, miss rates decreased as a function of rating quartile across all experiments and conditions. Table 1 shows the results of the permutation tests examining the LPE in each quartile and experiment. Importantly, with feedback present, there were significant LPEs in all four quartiles. Although the LPE in the 4th quartile is smaller than other quartiles, the difference in magnitude was not statistically significant for any quartile after correcting for multiple comparisons (*M*_diff_ < 0.2, *p* > 0.007; Bonferroni-corrected *α* = 0.003; see Additional file 1: Table S2). However, in both the third and fourth quartiles, when expressed as a proportion increase in miss rates due to low prevalence, the increase of miss rates under low prevalence is more than three times the miss rate under high prevalence (see Additional file 1: Fig. S1).

When participants could correct their responses, the LPE was significantly above zero in all quartiles except the fourth quartile (see Table 1), and the LPE was significantly smaller in the fourth compared to first quartile (*M*_diff_ = 0.31, *p* < 0.001), but none of the other quartiles differed significantly from any other quartile (*M*_diff_ < 0.2, *p* > 0.01). The LPE was non-significant in every quartile when trial-wise feedback was absent, and when only partial feedback was provided (see Table 1). When prevalence lowered to better match real road conditions, the LPE was only significant in the third quartile. However, in these experiments, the LPE trended towards the expected direction across all quartiles, and the LPE in the fourth quartile did not differ from the other quartiles (*M*_diff_ < 0.24, *p* > 0.05).

## Discussion

We examined whether perceived hazardousness, as assessed with continuous hazardousness ratings, modulated the low prevalence effect on road hazard detection. Overall, miss rates decreased as hazardousness ratings increased, consistent with the idea that hazards rated as less dangerous may be harder to detect compared to highly rated ones. In addition, when trial-wise feedback was provided, we found the LPE regardless of perceived hazardousness, suggesting that even extremely dangerous hazards are affected. Even for the most dangerous quartile of videos, miss rates for hazards at 4% prevalence are at least twice the miss rates for hazards at 50% prevalence (see Additional file 1: Fig. S1). These results suggest that high dangerousness alone is insufficient to alleviate the LPE.

Regression analyses showed that thresholds shifted towards the right under low prevalence conditions across all experiments. These results are consistent with prior studies suggesting that the LPE is a decisional effect rather than an attentional effect (Kosovicheva et al., 2023; Lyu et al., 2021; Wolfe et al., 2007). However, other mechanisms may also contribute to the LPE for detecting the most dangerous road hazards. For example, when participants were given the opportunity to correct their responses on each trial, we found reduced LPE for the most hazardous quartile of videos compared to the least hazardous quartile, suggesting that motor errors may be a potential contributor to the higher miss rate under low prevalence when feedback is present (Fleck & Mitroff, 2007; Van Wert et al., 2009). We found that giving participants an opportunity to correct their responses had the biggest effect for the most hazardous videos, suggesting that perhaps identifying hazards for the most highly rated videos may be an easier task than identifying hazards in more ambiguous videos (see Fig. 3). Although one may argue that motor errors are not true LPEs, these errors would still have grave consequences on the road (e.g., hitting the accelerator rather than the brake). In fact, on the road, drivers may not have enough time to correct such an error, which may lead to a collision.

In addition, our results did not show a PICC, wherein observers’ definition of a category (e.g., ‘hazard’) expands as instances of that category become rare (Levari et al., 2018). Since the LPE and PICC are opposite effects, Lyu et al. (2021) accounted for these differences by manipulating feedback; removing feedback shifted the criterion to be more liberal under low prevalence conditions (consistent with PICC), while including feedback made the criterion more conservative (consistent with the LPE). In contrast, we found no reversal of the LPE without feedback. In fact, although the LPE was not statistically significant in any quartile in the no-feedback experiment, the effects all tended towards a more conservative criterion under low prevalence, consistent with the LPE, suggesting that the PICC may not occur in this context. This discrepancy may come about because road situations are much more variable than stimuli that have been used to study the PICC. Recent work suggests that the criterion shift in PICC is sensitive to stimulus variability (Levari, 2022). Consistent with this idea, miss rates in the current dataset decreased with increased standard deviation and range of video hazardousness (see Supplementary Materials for more details). Road videos are extremely variable, which may counteract the PICC when feedback was absent. Future work may explore this by showing only the moderately dangerous hazards, which may counteract the LPE or could produce a PICC.

Despite it being extremely difficult to estimate the actual prevalence of hazards on the road, the hazard prevalence rates used in the current study are still substantially higher than what drivers would typically encounter in real driving (Guo et al., 2022). Even under the lowest prevalence (1%), drivers would have watched 10 hazard-present videos within an hour, whereas on the road, drivers would likely experience no collisions or near-collisions in a 1-h drive. These prevalences were used to ensure there were enough trials to estimate performance. Given these prevalences, the LPE is likely exacerbated in real driving. Moreover, in real driving situations, drivers often have much longer to respond to many hazards, as many eventual hazards on the road start off as latent hazards which are likely to unfold into hazardous situations but are not yet hazardous. Drivers likely look at and attend to latent hazards or may even choose to slow down or swerve to avoid them. However, the propensity of any one driver to do so likely depends on their tolerance for risk and their estimation of hazard likelihood. Our observers would have brought their own expectations to our study, which may be a caveat for generalizing our results to a real driving environment. Additionally, the consequences of missing hazards in the real world are catastrophic, which may reduce the LPE on the road.

Moreover, different road environments may have different prevalence rates for specific hazards. For example, cyclists are much more likely to appear on a busy urban road in the Netherlands compared to a rural road in the United States. Drivers respond to the relative prevalences of different kinds of hazards in simulation (Beanland et al., 2014), and likely also in real driving. Drivers likely develop expectations for the relative prevalences for certain kinds of hazards through gaining driving experience, but it is unclear how an unexpected or surprising hazard may affect its detection. Surprising events may capture attention, leading to higher detection performance, but they may also be subject to inattentional blindness (Wallisch et al., 2023), leading to lower detection performance, and it is unclear when each effect would occur in a driving context (Horstmann, 2015). How factors like context, expectation, and surprise affect hazard detection are potential avenues for better understanding the LPE for road hazards.

Although removing feedback reduced the LPE in the laboratory context, it is not a feasible intervention for combatting the LPE on the road because it is not possible to remove feedback during real driving. Feedback on the road can be catastrophic, resulting in a collision, and often self-generated by drivers, (e.g. “That was close”, “I didn’t need to brake”), and it is unclear whether these kinds of feedback affects hazard detection in the same way as those provided in the computerized task. Moreover, drivers need continuous visual input to drive, and removing the visual input that allows drivers to generate feedback would also remove visual information required to drive safely. For these reasons, future research need to investigate alternative interventions to combat the LPE on the road.

Overall, our analyses paint a disconcerting picture for the LPE in driving. Although it may seem intuitive that extremely dangerous scenarios should be immune to the LPE, our results suggest that this is not the case. Low prevalence inflates miss rates for extremely dangerous and less dangerous hazards alike. As roads become safer and hazards become rarer from efforts such as Vision Zero (Kim et al., 2017; Tingvall & Haworth, 1999), an international initiative to eliminate traffic fatalities and severe injuries, the remaining rare road hazards may be particularly difficult to address given the robustness of the LPE. Research and innovation efforts focusing on the most dangerous hazards are likely to have the greatest impact on road safety for the largest number of road users.

### Supplementary Information

**Additional file 1**. Full tables of statistical tests, as well as details of additional analyses on the LPE expressed as a proportional change, order effects, and effects of stimulus range and variability on error rate.

## Data Availability

The datasets supporting the conclusions of this article are available in the Open Science Framework repository. The hazard detection data are available at https://osf.io/r9uk7, and the ratings data and stimuli used in the study are available at https://osf.io/tgzb7.

## Abbreviations

LPE: Low prevalence effect
PICC: Prevalence induced concept change
