# Supplementary material for: Highly dangerous road hazards are not immune from the low prevalence effect
Source: Cogn Res Princ Implic. 2024 Feb 2;9:6. doi: 10.1186/s41235-024-00531-3 (PMC10834906; doi:10.1186/s41235-024-00531-3)
Supplement: Supplementary file 1 — Additional file 1. Full tables of statistical tests, as well as details of additional analyses on the LPE expressed as a proportional change, order effects, and effects of stimulus range and variability on error rate. [file 41235_2024_531_MOESM1_ESM.pdf]

Supplementary Materials for “Highly dangerous road hazards are not immune from the low prevalence effect“

Supplementary Table 1. Results of the general linear mixed effects model

**Fixed effects**	**Estimate**	**Standard Error**	***z***	***p***	**Odds Ratio (OR)**	**OR 95% CI**
Intercept	-3.88	0.14	-28.31	< 0.001	0.02	[0.02, 0.03]
Median Rating	8.74	0.16	54.52	< 0.001	6255.88	[4569.01,8565.52]
Low Prevalence	-1.50	0.06	-24.39	< 0.001	0.22	[0.20,0.25]
Expt Full Feedback	-0.07	0.19	-0.36	0.72	0.93	[0.64,1.36]
Expt Lower Prevalence	-1.87	0.18	-10.18	< 0.001	0.15	[0.11,0.22]
Expt No Feedback	0.62	0.19	3.28	0.001	1.86	[1.28,2.69]
Expt Partial Feedback	0.70	0.19	3.74	< 0.001	2.02	[1.40,2.93]
Median Rating : Low Prevalence	-1.53	0.19	-8.10	< 0.001	0.22	[0.15,0.31]
Median Rating : Expt Full Feedback	0.10	0.22	0.44	0.66	1.10	[0.71,1.71]
Median Rating : Expt Lower Prevalence	-0.04	0.23	-0.19	0.85	0.96	[0.61,1.51]
Median Rating : Expt No Feedback	-1.69	0.20	-8.36	< 0.001	0.19	[0.12,0.28]
Median Rating :	-0.87	0.21	-4.08	< 0.001	0.42	[0.28,0.64]
Expt Partial Feedback	-0.23	0.22	-1.06	0.29	0.80	[0.52,1.21]
Median Rating : Low Prevalence : Expt Lower Prevalence	1.11	0.23	4.80	< 0.001	3.04	[1.93,4.79]
Median Rating : Low Prevalence : Expt No Feedback	2.53	0.21	12.13	< 0.001	12.57	[8.35,18.93]
Median Rating : Low Prevalence : Expt Partial Feedback	1.09	0.21	5.14	< 0.001	2.97	[1.96,4.50]

Note: We fitted a binomial distribution to the data with a logit link function. The model formula was Participant Response ~ Median Rating + Prevalenct + Experiment + Median Rating : Prevalence + Median Rating : Experiment + Median Rating: Prevalence : Experiment + (1 | Participant). Experiment is abbreviated as Expt in the table. The Intercept represents the predicted rate of “hazard present” responses in the experiment allow participants to correct their response, at a high prevalence, and when the video has a median rating of 0.

Supplementary Table 2. Results of permutation tests examining whether LPE observed in the 4th quartile differs from those of other quartiles.

**Experiment**	**1 - 4**	***p***	**2 - 4**	***p***	**3 - 4**	***p***
Full Feedback	0.2	0.007	0.08	0.28	0.09	0.15
Response Correction	0.31	<0.001*	0.2	0.01	0.12	0.07
No Feedback	0.03	0.65	-0.06	0.31	-0.02	0.7
Partial Feedback	0.21	0.008	0.05	0.33	0.12	0.06
Lower Prevalence	0.08	0.57	0.04	0.79	0.24	0.05

* significant test according to a Bonferroni corrected alpha of 0.003.

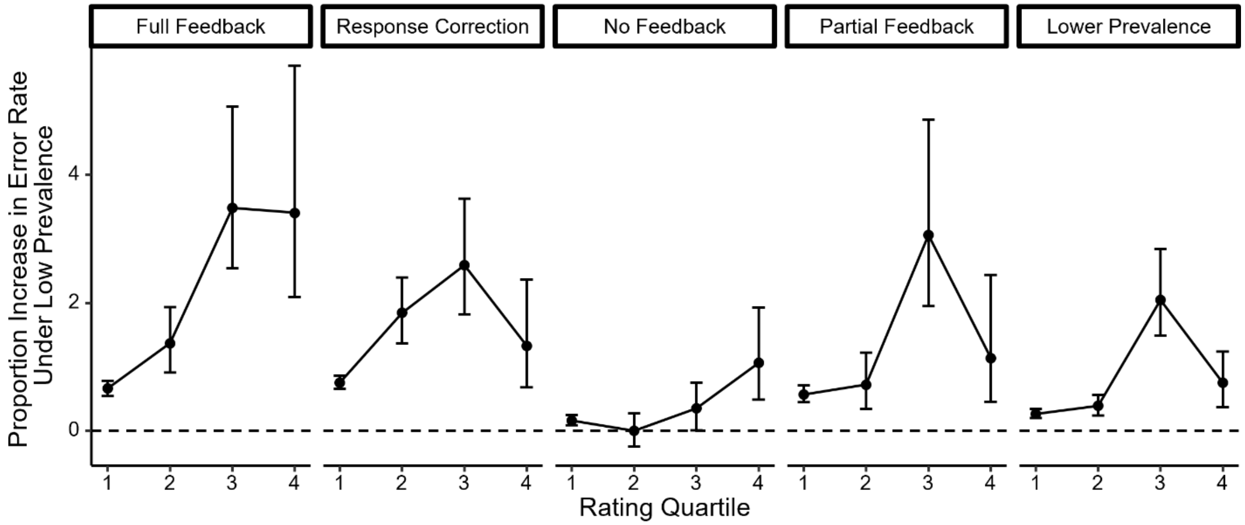

Supplementary Figure 1. The low prevalence effect in each experiment and quartile expressed as a proportion increase in error rate under low prevalence condition compared to the high prevalence condition (i.e. the difference in error rate between low and high prevalence divided by error rate under high prevalence). The horizontal dotted line represents no increase in error rate. Error bars represent bootstrapped 95% confidence intervals.

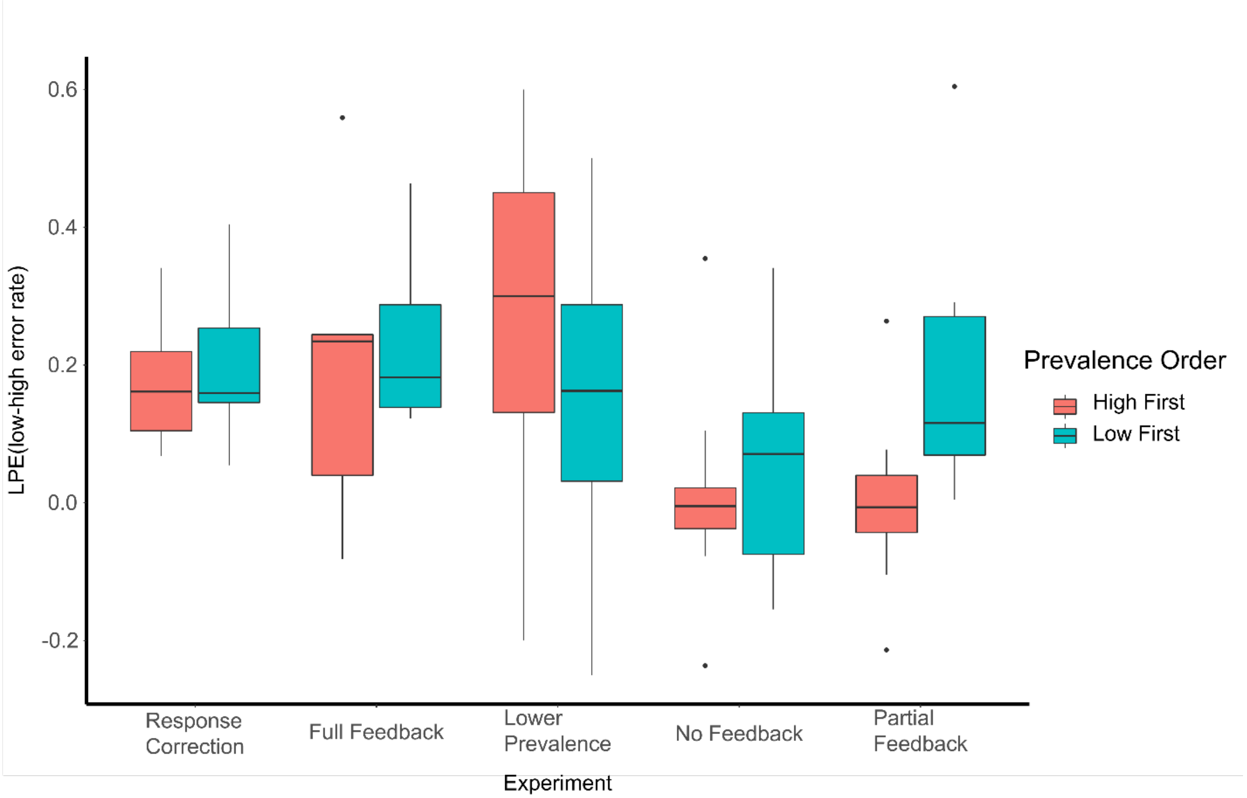

Supplementary Figure 2. The low prevalence effect in each experiment separated by the order in which participants completed the High and low prevalence sessions. Coral represents participants who completed the high prevalence condition first and turquoise represents participants who completed the low prevalence condition first. The low prevalence effect is the difference between the miss rates of hazards. A two-way ANOVA was conducted on the LPE with experiment and prevalence order as between-groups factors. There was a significant effect of experiment ($F(4,86) = 3.10$, $p = 0.02$, $\eta^2_{generalized} = 0.13$), but no significant effects of prevalence order ($F(1,86) = 0.66$, $p = 0.42$, $\eta^2_{generalized} = 0.01$), or the prevalence order and experiment interaction ($F(4,86 = 2.19$, $p = 0.08$, $\eta^2_{generalized} = 0.09$).

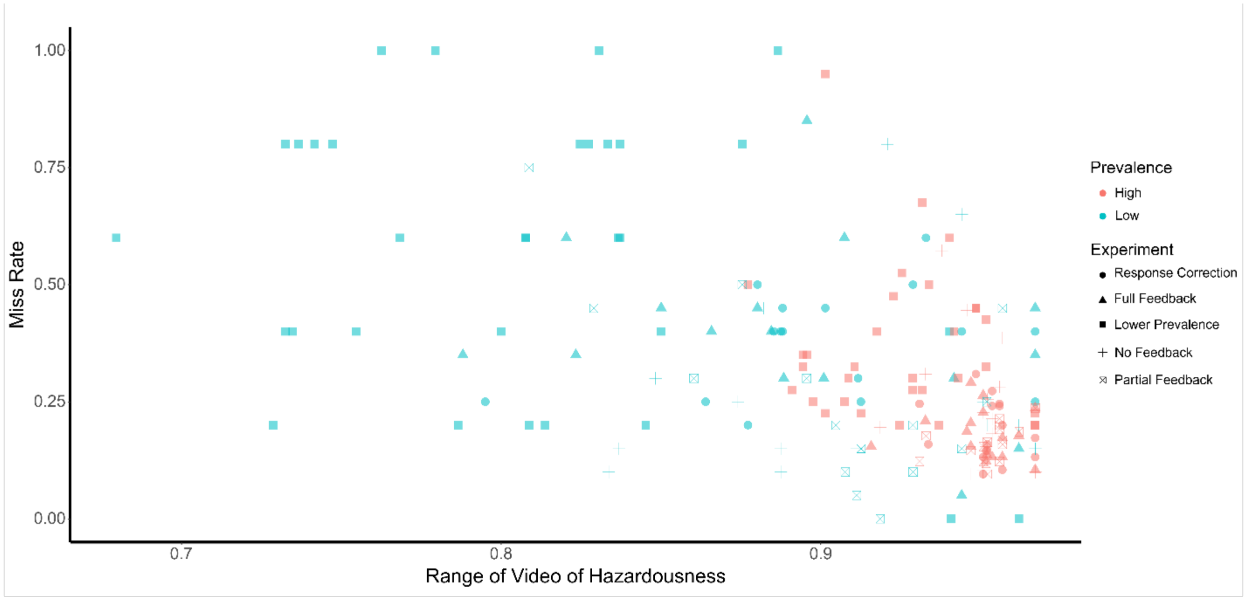

Supplementary Figure 3. Miss rate in a single session plotted as a function of the range of hazardousness of all videos shown within the session. Each point represents a single session. Coral and turquoise points represent high and low prevalence conditions respectively, and point shape represents experiment. There was an overall significant, negative relationship between miss rates and hazardousness range, and in each experiment except for the no feedback experiment (see Supplementary Table 3 for detailed statistics). These results are consistent with the idea that a larger range of stimuli is associated with lower miss rates.

Supplementary Table 3. Results of correlation tests conducted on miss rates and range of video hazardousness

**Experiment**	**Pearson's *r***	***t***	***p***	**Degrees of Freedom**	**95% Confidence Interval**
Response Correction	-0.35	-2.06	0.048	30	[-0.62, -0.004]
Full Feedback	-0.55	-3.64	0.001	30	[-0.76, -0.25]
Lower Prevalence	-0.44	-3.83	< 0.001	62	[-0.62,-0.22]
No Feedback	0.03	0.17	0.864	30	[-0.32,0.38]
Partial Feedback	-0.64	-4.55	< 0.001	30	[-0.81,-0.37]
Across Experiments	-0.53	-8.63	<0.001	190	[-0.63,-0.42]

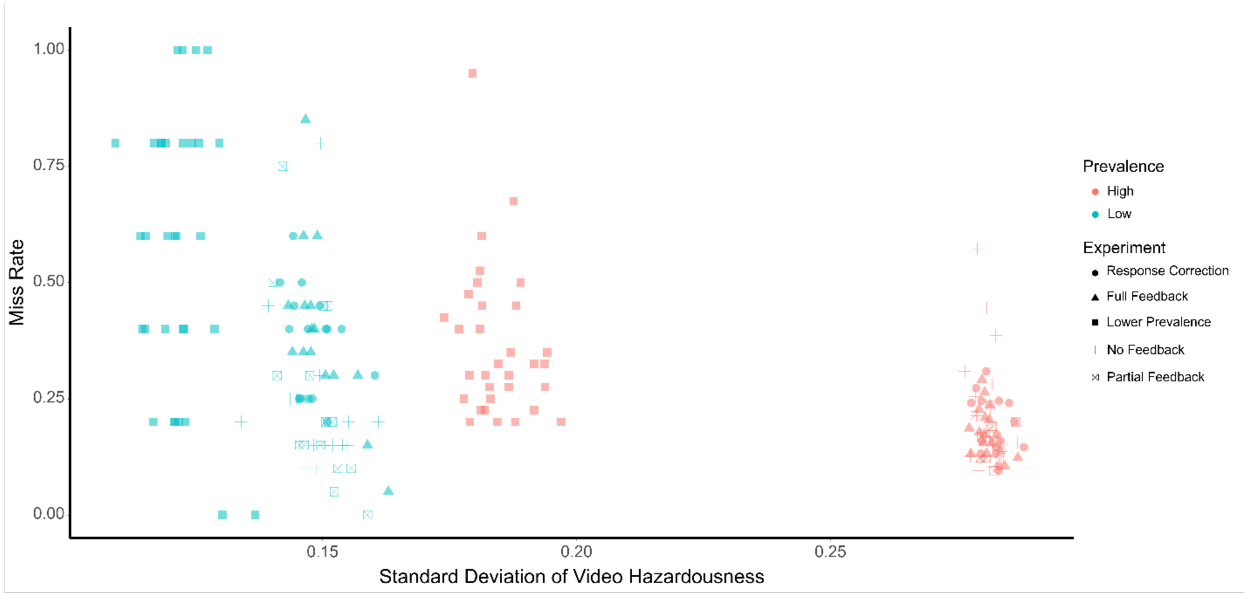

Supplementary Figure 4. Miss rates for each session are plotted as a function of the standard deviation of video hazardousness in the session. Figure conventions are identical to Supplementary Figure 3. There were significant negative correlations between miss rate and standard deviation of video hazardousness across experiments and in each experiment except for the no feedback experiment (see Supplementary Table 4 for detailed statistics). These results broadly align with the idea that increasing stimulus variability is associated with lower miss rates.

Supplementary Table 4. Results of correlation tests conducted on miss rates and the standard deviation of video hazardousness

**Experiment**	**Pearson's *r***	***t***	***p***	**Degrees of Freedom**	**95% Confidence Interval**
Response Correction	-0.74	-5.93	< 0.001	30	[-0.86,-0.52]
Full Feedback	-0.66	-4.84	< 0.001	30	[-0.82,-0.41]
Lower Prevalence	-0.42	-3.64	< 0.001	62	[-0.60,-0.19]
No Feedback	-0.10	-0.56	0.583	30	[-0.43,0.26]
Partial Feedback	-0.36	-2.11	0.044	30	[-0.63,-0.01]
Across Experiments	-0.5	-8.03	<0.001	190	[-0.60,-0.39]
